# Neuromuscular Junction Formation in Tissue-Engineered Skeletal Muscle Augments Contractile Function and Improves Cytoskeletal Organization

**DOI:** 10.1089/ten.tea.2015.0146

**Published:** 2015-09-03

**Authors:** Neil R.W. Martin, Samantha L. Passey, Darren J. Player, Vivek Mudera, Keith Baar, Linda Greensmith, Mark P. Lewis

**Affiliations:** ^1^ Arthritis Research UK Centre for Sport, Exercise and Osteoarthritis, National Centre for Sport and Exercise Medicine (NCSEM), School of Sport, Exercise and Health Sciences, Loughborough University, Loughborough, United Kingdom.; ^2^ Department of Pharmacology and Therapeutics, University of Melbourne, Parkville, Victoria, Australia.; ^3^ Institute of Orthopedics and Musculoskeletal Sciences, University College London, London, United Kingdom.; ^4^ Division of Neurobiology, Physiology and Behavior, University of California Davis, Davis, California.; ^5^ The Sobell Department of Motor Neuroscience and Movement Disorders, University College London, London, United Kingdom.; All work was performed at the Sobell Department of Motor Neuroscience and Movement Disorders, University College London, London, United Kingdom.

## Abstract

Neuromuscular and neurodegenerative diseases are conditions that affect both motor neurons and the underlying skeletal muscle tissue. At present, the majority of neuromuscular research utilizes animal models and there is a growing need to develop novel methodologies that can be used to help understand and develop treatments for these diseases. Skeletal muscle tissue-engineered constructs exhibit many of the characteristics of the native tissue such as accurate fascicular structure and generation of active contractions. However, to date, there has been little consideration toward the integration of engineered skeletal muscle with motor neurons with the aim of neuromuscular junction (NMJ) formation, which would provide a model to investigate neuromuscular diseases and basic biology. In the present work we isolated primary embryonic motor neurons and neonatal myoblasts from Sprague-Dawley rats, and cocultured the two cell types in three-dimensional tissue-engineered fibrin hydrogels with the aim of NMJ formation. Immunohistochemistry revealed myotube formation in a fascicular arrangement and neurite outgrowth from motor neuron cell bodies toward the aligned myotubes. Furthermore, colocalization of pre- and postsynaptic proteins and chemical inhibition of spontaneous myotube twitch indicated the presence of NMJs in the innervated constructs. When electrical field stimulation was employed to evoke isometric contractions, maximal twitch and tetanic force were higher in the constructs cocultured with motor neurons, which may, in part, be explained by improved myotube cytoskeletal organization in these constructs. The fabrication of such constructs may be useful tools for investigating neuromuscular pharmaceuticals and improving the understanding of neuromuscular pathologies.

## Introduction

Neuromuscular junctions (NMJs) are a highly specialized synapse in the peripheral nervous system, which regulate skeletal muscle contraction, and consist of a presynaptic motor neuron terminal, synaptic cleft, and postsynaptic motor end plate.^1^ A number of neuromuscular and neurodegenerative diseases impact upon the integrity of the NMJ either at the presynaptic or postsynaptic membrane, and consequently lead to loss of muscle mass and muscle weakness.^2^ Furthermore, in aged individuals, degradation of acetylcholine receptors (AChRs) on the postsynaptic membrane can lead to denervation and subsequently the age-related loss of muscle size and function.^3^ Current methodologies for investigating such neuromuscular pathologies rely heavily on the use of animal models such as transgenic mice,^4^ which have inherent sampling and ethical implications, or monolayer cell cultures,^5^ which fail to mimic the *in vivo* niche and are limited by the inability to measure muscle function and phenotype.

By contrast, tissue engineering techniques can be used to generate biomimetic model tissues in three-dimensions (3D), which better resemble native tissues and can be used for physiological, clinical, and pharmaceutical testing with fewer ethical issues. Indeed, engineered constructs display many of the same morphological characteristics as *in vivo* skeletal muscle, such as the development of aligned myotubes, which are orientated in parallel to one another and are surrounded by a biological matrix.^6,7^ Furthermore, engineered muscles are capable of active contractions in response to electrical stimuli, and exhibit positive force-frequency and accurate length-tension relationships.^8^ However, although the field of skeletal muscle tissue engineering is becoming ever more advanced, little data exist with regard to the integration of other cell types which are fundamental to skeletal muscle structure and function *in vivo*. In particular, the addition of motor neurons toward the goal of generating NMJs has been sparsely considered.

Neuronal input is essential for optimal muscle development and function. The absence of innervation during embryonic development has been shown to result in the degeneration of myotubes,^9^ ablation of secondary myotube development,^10^ and reduction in myotube size.^11^ Furthermore, slow myosin heavy chain expression is reduced in the absence of innervations,^11^ which in turn will affect muscle phenotype. In adult rodents, neurotomy results in immediate reductions in contractile force,^12^ and in spinal cord injured humans there is a quickening of the contractile kinetics. Therefore, it is clear that the presence of motor neurons and functioning NMJs regulate fiber size, type, and maturation. As such, an engineered muscle, which is innervated by motor neurons, would be expected to better represent *in vivo* tissue by means of improved structure and function. Moreover, engineered skeletal muscle with a motor neuron interface and NMJs would not only generate a more physiologically relevant tissue, but could also be used to help understand the biology of the NMJ in health and disease, as well as provide a platform to assess pharmaceutical treatments.

To date, the majority of investigations concerned with the generation of NMJ's *in vitro* have been carried out using monolayer culture techniques. Indeed, combinations of rodent and human-derived myotubes and motor neurons cocultured on functionalized glass coverslips have resulted in NMJ generation as evidenced by immunolabeling of neuromuscular proteins and chemical inhibition of neuron-mediated myotube contractions.^13–15^ Furthermore, Southam *et al.* recently reported the use of microfluidic technologies to promote axonal growth in a directed manner toward myotube cultures, resulting in neuromuscular interactions and apparent NMJ formation.^16^ However, skeletal muscle 3D tissue-engineered constructs have generally been innervated in a poorly defined manner. Spontaneous myotube contractions and advanced differentiation have been observed as a result of culturing rodent spinal cord slices within a 3D myoblast-seeded gel.^17^ Larkin and colleagues engineered laminin-based skeletal muscle constructs and cocultured them with spinal cord explants, witnessing some alterations in myosin heavy chain expression and augmented force production. Alternatively, engineered muscle constructs have been implanted *in vivo* in close proximity to severed nerves causing the nerve to reattach to the muscle construct,^18,19^ leading to improved contractile force production following electrical stimulation. Recently, neural stem cells have been cocultured in a systematic manner on top of hydrogels seeded with the myogenic C2C12 cell line, showing colocalization of NMJ proteins and responsiveness to chemical neural stimulation^20^ and the addition of PC12 neural cells to C2C12's improved levels of differentiation and myogenic gene expression in 3D gelatin-based hydrogels.^21^ However, the development of a defined and isolated 3D tissue-engineered neuromuscular interface generated from primary cells, which is structurally and physiologically similar to the mammalian synapse, has yet to be established.

The aim of the present work was to isolate primary embryonic rodent motor neurons and coculture them alongside primary neonatal rodent myoblasts in an established tissue-engineered skeletal muscle model,^6,22^ and subsequently identify if NMJs were present and if the structure and function of the construct were improved. Indeed, we demonstrate in this study that the addition of motor neurons to engineered skeletal muscle leads to observable neuromuscular interactions, which can be manipulated chemically, suggesting the presence of NMJs. In addition, motor neuron coculture leads to the generation of a muscle tissue more similar to native skeletal muscle by virtue of improved functionality and augmented myotube morphology. Models such as this may be used to advance the understanding of neuromuscular degeneration and diseases in the near future.

## Materials and Methods

### Isolation of primary muscle-derived cells

Primary muscle-derived cells (MDCs) were isolated from neonatal (P1) Sprague-Dawley rats as previously described^7^ in accordance with the code of practice for the humane killing of animals under Schedule 1 of the Animals (Scientific Procedures) Act 1986. Briefly, skin was removed from the hindlimbs of P1 pups and the underlying tissue transferred to a Petri dish containing phosphate-buffered saline (PBS; Sigma-Aldrich, Dorset, United Kingdom) supplemented with 2% penicillin (100 units/mL)/streptomycin (100 μg/mL) (P/S) (Gibco/Invitrogen, Paisley, United Kingdom). The muscle tissue was separated from the bone, and the remaining muscle tissue was suspended in a 0.1% collagenase solution (Gibco/Invitrogen) and transferred to a shaking incubator set to 300 rpm for 50 min, with occasional trituration by pipetting.

The resulting cell suspension was passed through a 100 μm and then a 40 μm mesh filter (BD Biosciences, East Rutherford, NJ) to remove any debris or undigested tissue fragments, before being spun at 480 *g* for 10 min. The cells were resuspended in 2 mL growth medium (GM) consisting of high glucose Dulbecco's modified Eagle's medium (Gibco/Invitrogen), supplemented with 20% fetal bovine serum (PAA, Somerset, United Kingdom), and 1% P/S.

To characterize the isolated cell population, primary MDCs were plated on to 0.2% gelatin-coated coverslips at a density of 10 × 10^4^ cells/cm^2^. Cells were fixed after 3 days in culture and subsequently stained for the myogenic protein Desmin (see immunohistochemistry). It was found that 32% ± 5% of the isolated cells were myogenic in nature.

### Isolation of primary mixed ventral horn-derived cells

Primary motor neurons were isolated from rat embryos at gestational age E14. Pregnant Sprague-Dawley females were euthanized by exposure to a rising concentration of carbon dioxide gas, in accordance with the code of practice for the humane killing of animals under Schedule 1 of the Animals (Scientific Procedures) Act 1986.

Embryos were removed following hysterectomy and transferred to a Petri dish containing Hank's balanced saline solution (HBSS; Sigma-Aldrich) supplemented with 2% P/S. Spinal cords were separated from the surrounding tissue and the meninges carefully removed. The dorsal horn was then cut away from the ventral portion of the spinal cord and discarded. The ventral horns from individual embryos were pooled and incubated in a 0.025% trypsin solution (type XII-S; Sigma-Aldrich) in HBSS for 10 min. They were then transferred to a fresh solution containing 800 μL L-15 medium (Gibco/Invitrogen), 100 μL 4% bovine serum albumin (BSA; Sigma-Aldrich), and 100 μL DNAse (1 mg/mL; Sigma-Aldrich). The spinal cords were agitated vigorously until they had disaggregated and were then triturated and left to settle. After 2 min, the solution was transferred to a 15-mL centrifuge tube (care was taken to avoid transferring any undissociated fragments). This process was repeated on two more occasions, and the three supernatants were pooled before being spun through a 1 mL 4% BSA cushion for 5 min at 370 *g*. Once the supernatant had been removed, the pellet was resuspended in 1 mL of complete neurobasal medium (CNB) consisting of neurobasal medium (Gibco/Invitrogen) supplemented with 2% B27 supplement (Gibco/Invitrogen), 2% horse serum (PAA), 1% P/S, 0.1 ng/mL glial cell derived neurotrophic factor (Alomone Labs, Jerusalem, Israel), 0.5 ng/mL ciliary neurotrophic factor (Alomone Labs), 0.1 ng/mL brain derived neurotrophic factor (Alomone Labs), 0.05% 2-mercaptoethanol (Gibco/Invitrogen), and 0.025% 200 mM glutamine (Gibco/Invitrogen).

The isolated ventral horn-derived cells were plated on to coverslips, which had previously been coated with 1.5 μg/mL polyornithine and 3 μg/mL laminin, at a density of 37.5 × 10^4^ cells/cm^2^ to characterize the cell population. Cells were fixed after 3 days and immunostained for MAP-2 (see immunohistochemistry) as marker of neuronal commitment. It was found that 35% ± 2% of the cell population were MAP-2 positive.

### Tissue engineering 3D fibrin-based myotube–motor neuron constructs

**FIG. 1.**
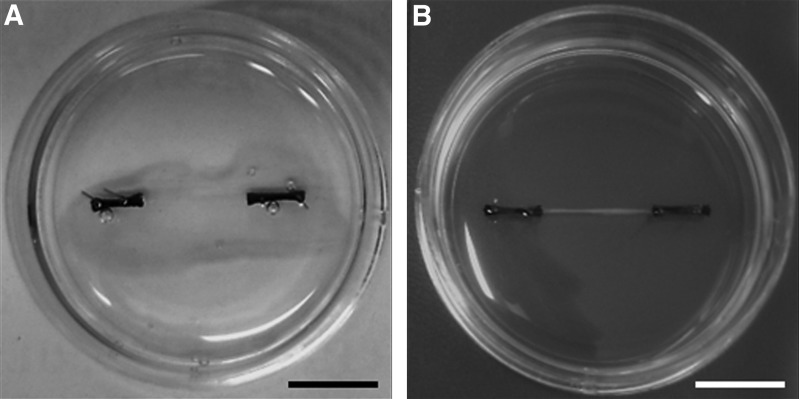
Macroscopic images of tissue-engineered skeletal muscle constructs at 4 and 18 days in culture. **(A)** By 4 days in culture at the time of motor neuron seeding the gel has begun to self-assemble, but still occupies a large surface area allowing the neurons to attach. **(B)** At the end of the culture period the constructs are completely assembled into a three-dimensional (3D) structure, in which the motor neurons and myotubes are encapsulated. Scale bars = 10 mm.

Fibrin gels were prepared as previously described.^6^ Briefly, two 6 mm silk sutures were pinned into Sylgard-coated 35-mm plates 12 mm apart using 0.15 mm minutien pins (Entomoravia, Slavkov u Brna, Czech Republic). Plates were sterilized by ultraviolet light and washing with 70% ethanol and left to dry for 3 h. Each plate then received 500 μL of GM containing 10 U/mL thrombin (Sigma-Aldrich) and 80 μg/mL aprotinin (Sigma-Aldrich) which was spread evenly over the surface of the plate ensuring the sutures were fully covered. Two hundred microliters of 20 mg/mL stock fibrinogen (Sigma-Aldrich) solution was then added to the plate, and was agitated gently to ensure even distribution and then left to incubate for 10 min at room temperature before being transferred to the incubator (37°C) for 1 h. Once the gel had set, 2 × 10^5^ primary MDCs were evenly plated on top of the gel surface in 2 mL of GM containing 0.25 mg/mL 6-aminocaproic acid (Sigma-Aldrich) to prevent fibrin degradation. Once MDCs within the gels became confluent (typically 4 days of culture), 5 × 10^5^ primary ventral horn cells were plated on to the gel surface and allowed to attach for 10–15 min before 2 mL of CNB supplemented with 0.25 mg/mL 6-aminocaproic acid, 10 ng/mL insulin-like growth factor 1 (AbD Serotec, Oxford, United Kingdom), and 200 ng/mL recombinant rat Agrin (R&D Systems, Abingdon, United Kingdom) was added. Culture medium was replaced every other day for the duration of experimentation. It is of note that the time at which ventral horn-derived cells were seeded on to the fibrin gel (Fig. 1A) dictated that cells which adhered to the substrate would be encapsulated within the fully formed 3D construct (Fig. 1B), and, therefore, be in close proximity to the myotubes throughout the core of the tissue.

### Immunohistochemistry

Constructs were washed twice with PBS and then fixed using ice-cold methanol and acetone. Subsequently, 3D constructs were cut away from the sutures and placed on polylysine-coated microscope slides (VWR, Leicestershire, United Kingdom) and ringed with PAP pen (Dako, Cambridgeshire, United Kingdom). Constructs were blocked with 1× Tris-buffered saline (TBS; 0.5 M) containing 5% serum (Sigma-Aldrich) and 0.2% Triton X-100 (Fisher Scientific, Leicestershire, United Kingdom) for 90 min. Following three washes with TBS, constructs were incubated overnight with primary antibodies in a humidified staining chamber. Myotubes and motor neurons were identified with mouse anti-Desmin (Clone D33; Dako) and rabbit anti-MAP-2 (Millipore, Herefordshire, United Kingdom), respectively, both diluted 1:200 in TBS. Presynaptic nerve terminals were identified using antisynaptic vesicle protein-2 (SV-2; DSHB, Iowa City, IA) diluted 1:10 in TBS. After overnight incubation, constructs were washed three times in TBS and incubated for 3 h with goat anti-mouse AlexaFluor 488 and donkey anti-rabbit AlexaFluor 568 secondary antibodies (Invitrogen) both diluted 1:200 in TBS, and 4′,6-diamidino-2-phenylindole (DAPI; Sigma-Aldrich) to visualize nuclei. AChR's were stained using Texas red-conjugated α-Bungarotoxin (Sigma-Aldrich) diluted 1:1000 during the secondary antibody incubation. Following three further washes in distilled water, constructs were mounted on glass coverslips using a drop of Fluoromount™ (Sigma-Aldrich) mounting medium.

Three-dimensional constructs were imaged using a Zeiss LSM-710 confocal microscope (Zeiss, Cambridgeshire, United Kingdom) and were analyzed using ImageJ software (NIH, Bethesda, MD). Fusion index was calculated as the number of nuclei incorporated into myotubes expressed as a percentage of the total number of Desmin-positive nuclei and striated myotubes were considered to be those which exhibited a striated pattern at any point along their length.

### RNA extraction and real time polymerase chain reaction

Media was removed from plates and constructs were washed twice with PBS before the addition of 500 μL of TRIzol^®^ (Life Technologies, Paisley, United Kingdom). Each construct was then homogenized and the RNA extracted according to the manufacturer's instructions. Reactions were prepared in triplicate in 96-well plates and consisted of 70 ng of RNA diluted in 9.5 μL of nuclease-free water, 0.15 μL of both forward and reverse primers at a final concentration of 750 nM (see Table 1 for primer sequences), 0.2 μL of the QuantiFast Reverse Transcriptase Kit (Qiagen, Crawley, United Kingdom), and 10 μL of SYBR Green Mix (Qiagen). Plates were transferred to an Mx3005P Thermal Cycler for one-step RT-PCR, which was programmed to perform the following: 10 min at 50°C (reverse transcription), 5 min at 95°C (Hot Start Taq polymerase), followed by 40 cycles of 95°C for 10 s and 60°C for 30 s. Fluorescence was detected at the end of each cycle and data were analyzed using the 2^(−ΔΔCT)^ method using POLR2β as a reference gene and a single aneurally cultured construct from each experiment as a calibrator.

**Table 1.** Primer Sequences Used for Detection of Myosin Heavy Chain mRNA Levels

*Target mRNA*	*Primer sequence 5′-3′*	*NCBI reference*
MYH3	F: CAAGTTCATCCGCATCCAT	NM_012604.1
	R: TCGTAAGGGTTGGTCGTAA	
MYH8	F: TACGCCAGTGCTGAAGCAGGTA	NM_001100485.1
	R: CCATGGCACCGGGAGTTTTCG	
MYH1	F: CCTAAAGGCAGACTCTCCCACTGGG	NM_001135158.1
	R: GGCCATCTCGGCGTCGGAAC	
MYH7	F: AAGAAGGTGCGCATGGACCTGG	NM_017240.2
	R: CCTGCTCATCCTCAATCCTGGCG	
POLR2β	F: ACATAACGAAGACGGTCAT	NM_001106002.1
	R: TAAGCCATTCAACAAGCAATA	

### Evaluation of chemical inhibition of myotube contraction

One anchor from the fibrin construct to be analyzed was removed from the Sylgard and attached to a model 403A force transducer (Aurora Scientific, Dublin, Ireland) using canning wax. The force transducer was connected to a PowerLab 4/25T unit with associated software (ADInstruments, Oxford, United Kingdom), and force generated by the constructs was measured at 1 kHz. The baseline force generated by the constructs (spontaneous twitch) was recorded for 60 s, before 100 μM d-tubocurarine (Abcam, Cambridge, United Kingdom) was added to the medium bathing the constructs. Recording continued after application of the drug for up to 3 min. Twitch frequency at baseline and following the addition of d-tubocurarine was generated from analyzing the initial 1 min of force and the second minute of contractile force following d-tubocurarine treatment, respectively. The second minute of force after d-tubocurarine addition was selected to discount any measurement of construct movement due to the physical addition of the drug solution to the bath medium.

### Assessment of muscle function by electrical stimulation

Constructs were washed twice in PBS and connected to a force transducer as described above. Three milliliters of Krebs–Ringer–Hepes (KRH; 10 mM HEPES, 138 mM NaCl, 4.7 mM KCl, 1.25 mM CaCl_2,_ 1.25 mM MgSO, 5 mM glucose, 0.05% BSA in dH_2_0) buffer solution was then added to the dish containing the construct and two stainless steel electrodes were placed in position either side of the construct and submerged in the KRH buffer before functional testing. Impulses were generated using LabVIEW software (National Instruments, Berkshire, United Kingdom) connected to a custom-built amplifier. Data were acquired using PowerLab software (ADInstruments) with a sampling rate of 1 kHz.

Rheobase was calculated as the electric field strength eliciting 50% peak twitch force with a 1.2 ms pulse width, and maximal tetanic force was determined using a 100 Hz impulse train at 3.5 V/mm. Time to peak twitch (TPT) was defined as the amount of time taken for maximal twitch force to raise from baseline to peak, and half relaxation time (HRT) was the amount of time taken for the twitch amplitude to fall by half at the cessation of stimulation. Fatigue was tested by eliciting tetanic contractions at 50 Hz for 3 min with a 1 s rest between pulse trains, and the force decrement between the first and last contraction was calculated.

### Statistical analysis

All data are presented as mean ± standard deviation, unless otherwise stated. Mann–Whitney *U* tests were used to determine if statistical differences existed between constructs cultured with or without motor neurons. The results of the chemical inhibition study were analyzed using a two-way mixed measure ANOVA. All statistical analyses were conducted using SPSS version 22.0.

## Results

### Motor neuron–myotube interactions

**FIG. 2.**
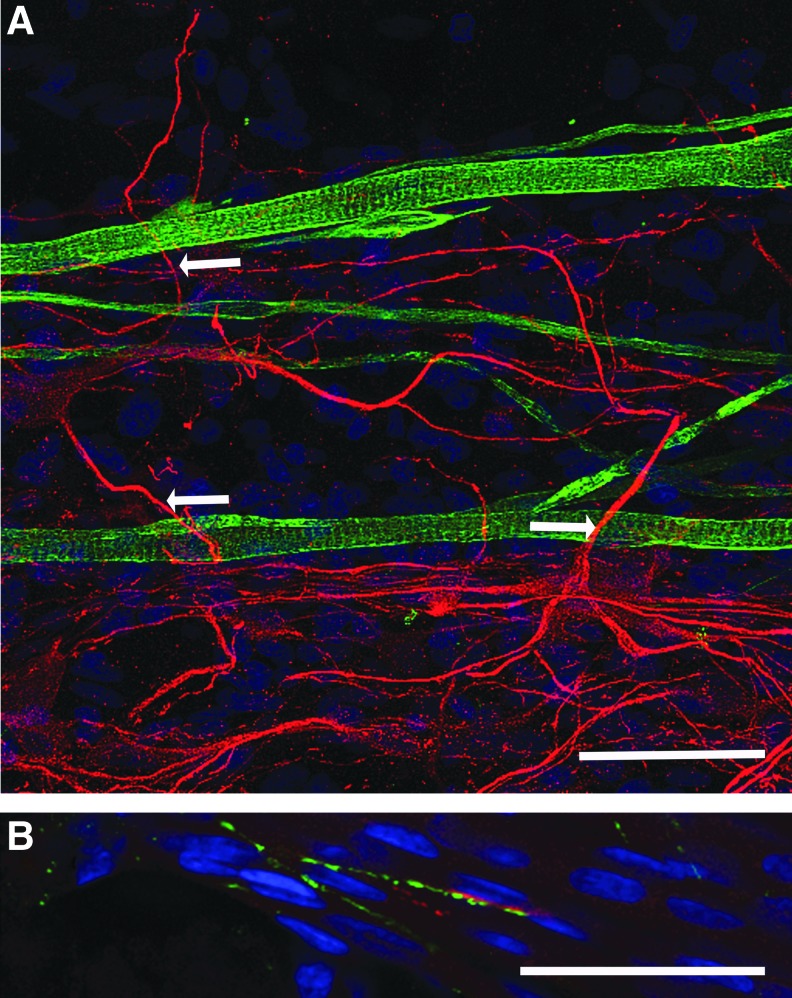
Primary myotubes and motor neurons coexist and interact in 3D tissue-engineered constructs. **(A)** Immunostaining for Desmin (*green*) and MAP-2 (*red*) labels myotubes and motor neurons, respectively, and shows uniaxial alignment of myotubes and deviation of neurites toward the myotubes. **(B)** Colocalization of acetylcholine receptor (AChR; *red*) and synaptic vesicle protein-2 (SV-2; *green*) marks the colocalization of pre- and postsynaptic membranes on the myotube and motor neuron, respectively. Cells are counterstained with 4′,6-diamidino-2-phenylindole (DAPI; *blue*). Scale bars = 50 μm. *Arrows* indicate the deviation of neurites away from the direction of mechanical signals.

Immunohistochemical analysis of cocultured constructs demonstrated uniaxial alignment of myotubes within the 3D hydrogels in response to the mechanical cues generated by the opposing anchor points. Furthermore, MAP-2 staining of motor neurons confirmed the adherence of the ventral horn-derived cells to the fibrin gel substrate, and neurites extended away from the cell body in a manner characteristic of this cell type (Fig. 2A). Interestingly, these neural projections were frequently observed extending toward myotubes (Fig. 2A arrows), perpendicular to the alignment of the majority of cellular structures, suggesting that myotube–motor neuron interactions were occurring within the 3D constructs, and these interactions could override the mechanical signals inherent to the system.

To investigate this interaction further, immunostaining for presynaptic (SV-2) and postsynaptic (AChR) structures was conducted to observe colocalizations as an indication of potential NMJ formation. Colocalization between SV-2 and AChR was found to occur (Fig. 2B), suggesting that there was putative NMJ formation within cocultured fibrin constructs, despite the frequency of interaction being relatively low (0.22 ± 0.06/mm^2^). No SV-2 staining was detected in constructs cultured in the absence of motor neurons.

### Chemical inhibition of contractile activity

**FIG. 3.**
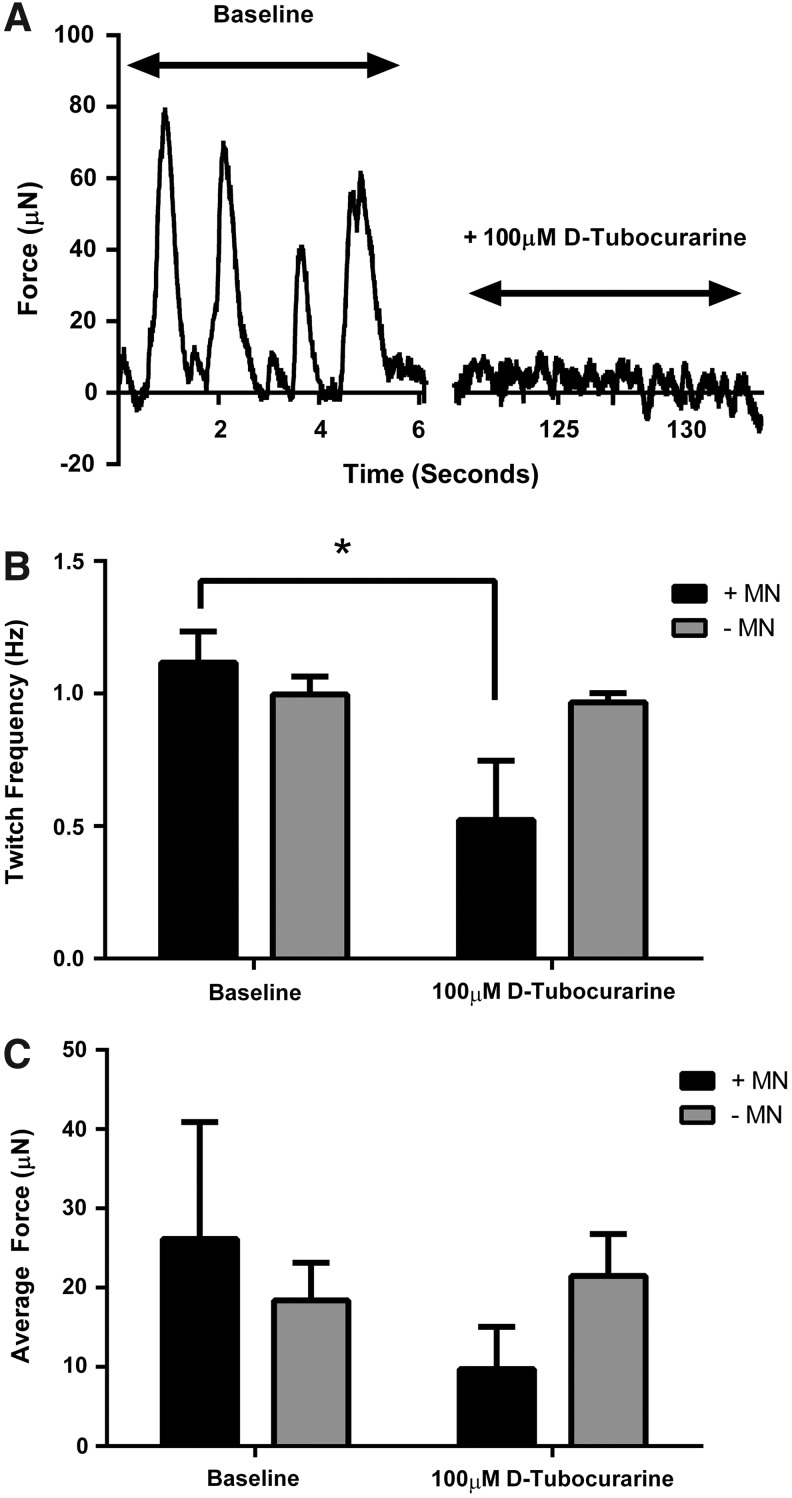
Chemical antagonism of the AChR using d-tubocurarine indicates the presence of neuromuscular interactions in tissue-engineered skeletal muscle–motor neuron cultures. **(A)** Representative force trace showing spontaneous twitch at baseline and in the presence of 100 μM d-tubocurarine. **(B)** Twitch frequency is reduced by the addition of d-tubocurarine only in cocultured constructs. **(C)** Average force is reduced in cocultured constructs following the addition of d-tubocurarine, but not in aneural constructs. Data are mean ± standard deviation (SD) for *n* = 3 constructs in each condition. * Indicates a statistically significant difference (*p* ≤ 0.05).

Following 18 days of culture (14 days coculture), no statistically significant difference existed in spontaneous myotube twitch amplitude (*p* = 0.22) or frequency (*p* = 0.10) between muscle constructs cultured with or without motor neurons (Fig. 3B, C), although the mean amplitude and frequency tended to be higher in cocultured constructs. The addition of the neuromuscular blocking agent d-tubocurarine, however, caused reductions in both twitch frequency (interaction effect *p* ≤ 0.05) and twitch amplitude (interaction effect *p* = 0.07) only in those constructs which were cultured with motor neurons (Fig. 3A–C). This suggests that there is a degree of functional synaptic contact in the myotube–motor neuron constructs, which can be manipulated chemically.

### Electrically evoked contractile properties

**FIG. 4.**
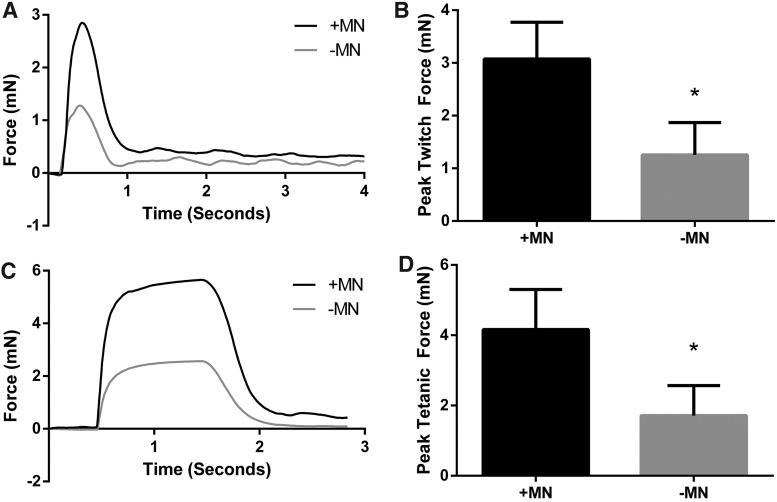
Contractility of tissue-engineered skeletal muscle is improved by coculture with motor neurons. **(A, B)** Twitch force is augmented by coculture with motor neurons. **(C, D)** Maximal isometric force is greater when myotubes are cocultured alongside motor neurons. Data are mean ± SD for *n* = 3 constructs in each condition. * Indicates statistically less than +MN (*p* ≤ 0.05).

Electrical field stimulation was utilized to investigate the effect of neuronal presence on the contractile characteristics of engineered skeletal muscle. After 18 days *in vitro*, constructs cultured with motor neurons exhibited ∼145% greater twitch force when maximally stimulated compared to aneural controls (3.07 ± 0.70 mN vs. 1.25 ± 0.61 mN, *p* ≤ 0.05, Fig. 4A, B). Similarly, when a 1 s pulse train was delivered to the constructs to evoke a fused tetanic contraction, force production was augmented by ∼143% in cocultured constructs in comparison to constructs cultured in the absence of neurons (4.16 ± 1.14 mN vs. 1.71 ± 0.85 mN, *p* ≤ 0.05, Fig. 4C, D), thus clearly indicating a positive effect of motor neuron coculture on the ability of skeletal muscle constructs to maximally contract.

**FIG. 5.**
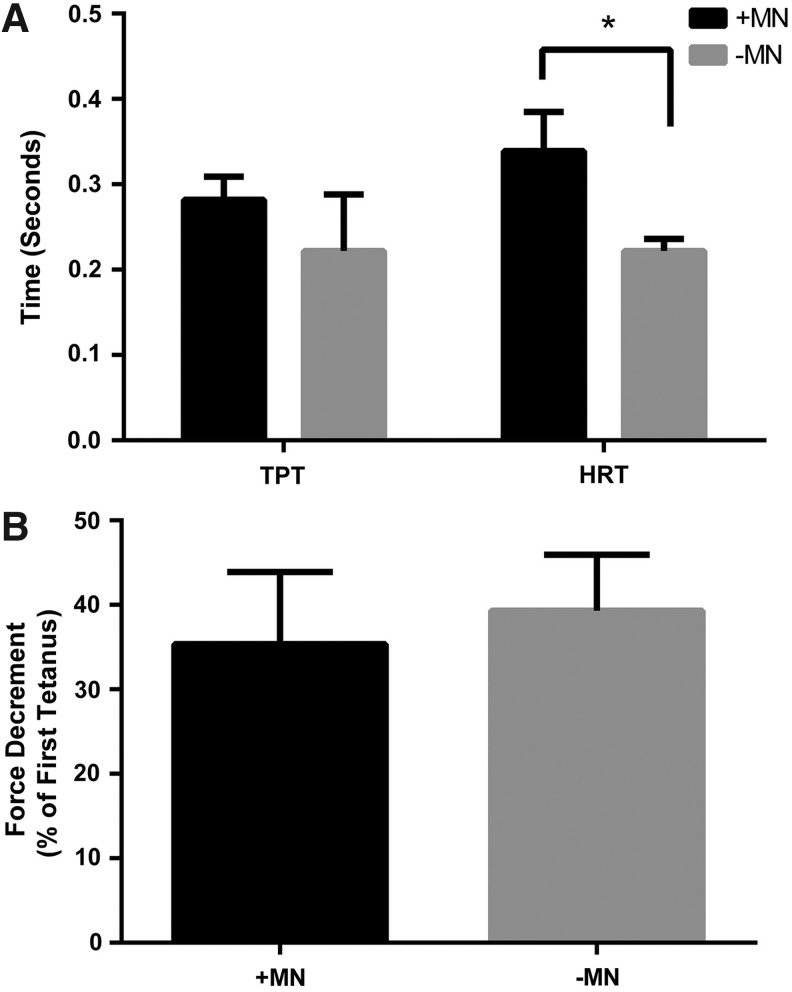
The addition of motor neurons to engineered skeletal muscle constructs effects markers of muscle phenotype. **(A)** Time to peak twitch and half relaxation time are increased in cocultured versus aneural constructs. **(B)** Force decrement over a 3-min fatigue test was unaffected by the addition of motor neurons to engineered skeletal muscle. Data are mean ± SD for *n* = 3 constructs in each condition. * Indicates a statistically significant difference (*p* ≤ 0.05).

Rheobase was used as a measure of muscle excitability, and did not appear to differ between constructs cultured in the presence or absence of motor neurons (0.40 ± 0.15 V/mm vs. 0.25 ± 0.13 V/mm, *p* = 0.15) suggesting that motor neurons did not affect this aspect of muscle function. HRT was, however, significantly increased by the addition of motor neurons to skeletal muscle 3D constructs (339 ± 46 ms vs. 222 ± 14 ms, *p* ≤ 0.05, Fig. 5A). Similarly, TPT was also somewhat affected by the addition of motor neurons and measured 282 ± 27 ms compared to aneural controls that measured 222 ± 66 ms, which although failed to reach statistical significance, represented a substantial effect (*p* = 0.20, *r* = 0.45, Fig. 5A). As such, motor neuron–myotube constructs appear to display a slowing of the contractile kinetics in comparison to constructs cultured in the absence of motor neurons.

To determine if the slowed twitch kinetics were matched by improvements in fatigue resistance (characteristics of a slow muscle) a 3-min fatigue test was conducted on constructs cultured in the presence and absence of motor neurons. However, the reductions in force over the course of the test were similar between conditions, with innervated constructs displaying a 35.35% ± 8.56% loss of force compared to a 39.35% ± 6.64% loss of force in aneural constructs (*p* = 0.50, Fig. 5B).

### mRNA expression of myosin heavy chain isoforms

**FIG. 6.**
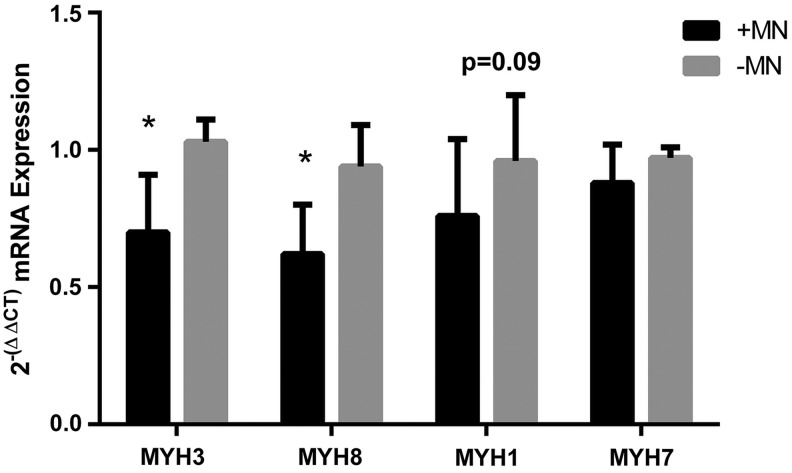
The mRNA expression of myosin heavy chain isoforms in 3D tissue-engineered skeletal muscle cultured in the presence or absence of motor neurons. Innervated constructs displayed reduced levels of *MYH3* (embryonic) and *MYH8* (perinatal) compared to aneural controls. The adult isoforms *MYH1* (fast) and *MYH7* (slow) not statistically different between groups. Data are mean ± SD for *n* = 8 constructs in each condition. * Indicates a statistically significant difference (*p* ≤ 0.05).

Since we observed some slowing of the contractile properties (HRT and TPT) when myotubes were cocultured with motor neurons, we looked to see if the expression of myosin heavy chain isoforms were altered in these constructs in comparison to controls (Fig. 6). Interestingly, we found that mRNA levels of *MYH3* and *MYH8*, which correspond to embryonic and neonatal isoforms, respectively, were significantly reduced in cocultured constructs (*MYH3*: 1.02 ± 0.08 aneural vs. 0.70 ± 0.21 innervated, *MYH8*: 0.94 ± 0.15 aneural vs. 0.62 ± 0.18 innervated, both *p* ≤ 0.05) after 18 days in culture/14 days coculture. There was also a somewhat reduced expression of the fast isoform *MYH1* that measured 0.73 ± 0.27 in innervated constructs compared to 0.96 ± 0.23 in aneural constructs, which although did not reach significance, represented a marked effect (*p* = 0.09, *r* = 0.44). However, expression of the slow isoform *MYH7* was maintained in cocultured constructs (0.97 ± 0.04 aneural vs. 0.88 ± 0.14 innervated, *p* = 0.12) This could indicate that myotubes cultured in the presence of motor neurons became slower in nature by virtue of maintenance of slow myosin heavy chain expression and concomitant reductions in all other isoforms.

### Myotube morphology

**FIG. 7.**
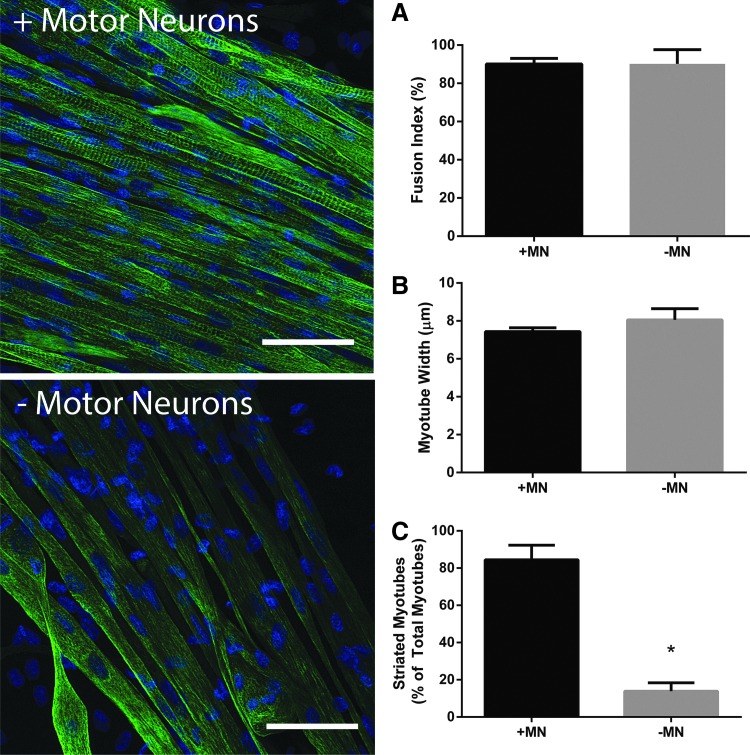
The presence of motor neurons in engineered skeletal muscle improves the myotube structure, but does not enhance levels of differentiation. Constructs immunostained for Desmin reveal that fusion index **(A)** and myotube width **(B)** are not influenced by the addition of motor neurons to 3D myotube cultures. However, the percentage of striated myotubes **(C)** is enhanced in cocultured versus aneural constructs. Cells are counterstained with DAPI (*blue*). Data are mean ± SD for *n* = 3 constructs in each condition. * Indicates statistically less than +MN (*p* ≤ 0.05). Scale bars = 50 μm.

To determine if any of the contractile differences observed between constructs cultured with or without motor neurons was due to alterations in morphology of the myotubes, 18-day-old constructs were immunostained for the cytoskeletal intermediate filament Desmin. Fusion index did not differ between innervated or aneural constructs (90.11 ± 2.89 vs. 90.17 ± 7.49, respectively, *p* = 0.35, Fig. 7A) and no difference existed in myotube number in each condition (14.87 ± 2.84 vs. 14.80 ± 1.11 myotubes per microscope frame in innervated and aneural cultures, respectively, *p* = 0.50) showing that the overall levels of differentiation were independent of neural input. Furthermore, myotube width, as an indicator of hypertrophy, was similar between conditions; with cocultured myotubes measuring 7.44 ± 0.20 μm and aneurally cultured myotubes measuring 8.06 ± 0.59 μm (*p* = 0.10, Fig. 7B). Strikingly, however, myotubes cocultured in 3D with motor neurons consistently presented with striations, whereas this striated pattern was rare in aneurally cultured myotubes. Indeed, when quantified, 84.42% ± 7.85% of myotubes were striated in the 3D cocultured constructs compared with only 13.90% ± 4.47% of myotubes in the aneural constructs (*p* ≤ 0.05, Fig. 7C). As such it appears that the presence of motor neurons has a maturation effect on myotubes in 3D hydrogels.

## Discussion

Neuromuscular disorders which impact upon both the motor neuron and skeletal muscle can lead to significant loss of muscle function and are often fatal.^2^ While there is much ongoing research in this area, there is a need to develop new methodologies that can reduce the reliance on animal use and accurately resemble the *in vivo* physiology of the neuromuscular interface. Tissue-engineered skeletal muscle has been consistently reported to exhibit structural and functional similarities to the native tissue^8^; however its integration with the peripheral nervous system has been scarcely studied. Indeed, the generation of an engineered tissue with NMJs would be a useful tool for physiologists and an ideal test bed to study neuromuscular therapeutics. In the present work we have produced an innervated 3D skeletal muscle from primary cells, which exhibits neuromuscular interactions, responsiveness to NMJ inhibitors, improved contractile function, and enhanced morphology, and as such represents significant advancement toward a fully integrated engineered *in vitro* skeletal muscle.

The attachment of embryonic rodent motor neurons to engineered skeletal muscle constructs was confirmed by immunostaining for MAP-2. Morimoto *et al.* recently identified that the addition of neurons to a 3D construct can be difficult since the cells can slip away from its surface. In the present work, the use of an established self-assembling tissue engineering method allowed the cells to attach to the substrate before it fully assembled, thereby contributing to improved cell attachment and allowing the motor neurons to be incorporated into the 3D scaffold. Furthermore, while the majority of MAP-2-positive cells adopted a longitudinal orientation within the constructs, frequently the neural extensions were seen tracking perpendicular to the lines of tension toward the myotubes (Fig. 1A). This differs from the data of Morimoto *et al.* who conducted alignment analysis on both myotubes and the cocultured neural stem cells, and showed that both cell types maintained longitudinal orientation. As such it appears that in the 3D myotube–motor neuron constructs described in this study, neurotrophic factors may predominate over mechanical signals, in turn encouraging neuromuscular interactions.

The utility of an engineered myotube–motor neuron construct is highly dependent upon the presence of functional NMJs. Identification of colocalized pre-and postsynaptic proteins in our models suggests that the interactions between motor neurons and myotubes did indeed result in NMJ formation. Furthermore, the addition of the AChR antagonist d-tubocurarine to the media bathing the constructs led to a reduction in twitch force and frequency in cocultured constructs, providing further evidence of functional NMJ formation in this model. Interestingly, spontaneous myotube twitch in aneural myotubes has previously been attributed to autocrine activation of AChR's by an ACh-like compound secreted by skeletal muscle myotubes.^23^ However, the present data, which show an effect of AChR blockade solely in constructs cocultured with motor neurons, perhaps suggests alternate mechanisms regulating spontaneous twitch in aneural and innervated myotubes, although the present study did not aim to elucidate these mechanisms. Putative NMJs have been previously described in 3D constructs as assessed by colocalized proteins^18,24^ and chemical contractile inhibition.^20^ However, this is the first report which has generated a 3D innervated skeletal muscle in a systematic, controllable manner utilizing primary cells. As such, this presents a significant advancement toward the utility of such a model in preclinical, physiological, and pharmaceutical testing.

It has previously been established that external electrical stimulation of myotubes in 3D constructs similar to those described in this study augments contractile force production.^25^ Similarly, coculture of 3D skeletal muscle constructs with spinal cord explant or implantation of engineered muscle in close proximity to severed nerves *in vivo* results in enhanced isometric tetanic force.^18,19,24^ In the present work both peak twitch and peak tetanic force were enhanced by the addition of motor neurons to skeletal muscle constructs, with peak force generation averaging ∼4 mN, more than an order of magnitude greater than the forces previously described in this or similar models.^24,26–28^ Thus, the addition of motor neurons to engineered skeletal muscle drives the maturation of the tissue, and results in a functional response closer to that of native skeletal muscle.

We further investigated the functional properties of the innervated constructs by assessing TPT, HRT, and rheobase as a measure of excitability. While the excitability of the tissue and TPT did not change, the HRT was significantly slowed in those constructs cocultured with motor neurons, suggesting that these constructs may have switched to a slow phenotype. Since slow muscles are fatigue resistant, we conducted a 3-min fatigue test on constructs cultured with or without motor neurons and found that the loss of force over the course of the test did not differ between conditions. In embryonic muscle development slow myosin heavy chain expression is lost when innervation is chemically ablated,^11^ and in cell culture models slow myosin heavy chain expression is dependent upon coculture with spinal cord explants.^29^ We, therefore, also conducted RT-PCR for myosin heavy chain isoforms in cocultured and aneurally cultured 3D skeletal muscle constructs. This analysis showed reduced expression of the developmental isoforms *MYH3* and *MYH8*, alongside small reductions in the fast isoform *MYH1*, with no observable changes in the slow isoform *MYH7*. This suggests that skeletal muscle constructs cultured with motor neurons may lose expression of more immature isoforms, while maintaining slow myosin heavy chain expression. Thus, overall analysis of the functional and mRNA data suggests that coculture of myotubes with motor neurons resulted in some features of a slow muscle, but this transformation was incomplete.

Since neural innervation during development can impact upon myotube size, number, and maturation,^9–11^ we finally sought to determine whether the presence of motor neurons in 3D skeletal muscle constructs effected myotube morphology, which may in turn explain the differences observed in contractile force generation. Interestingly, the presence of motor neurons had no effect on muscle precursor cell fusion nor did it induce any hypertrophic effect on the myotubes. However, Desmin immunostaining revealed a striated pattern in ∼85% of myotubes, which were cocultured alongside motor neurons while aneural cultures exhibited striations in only ∼13% of myotubes. Indeed, it has previously been shown that electrical stimulation can enhance the striations in C2C12 myoblasts cultured on electrospun scaffolds,^30^ and recently coculture of C2C12's with the PC12 neural cell line improved the levels of differentiation and myosin heavy chain mRNA expression in 3D hydrogels.^21^ It, therefore, seems likely that the presence of motor neurons induced a maturation effect on the myotubes through enhanced cytoskeletal organization, which in turn is responsible, at least in part, for the improved force generation in these constructs.

## Conclusions

The development of tissue-engineered skeletal muscle constructs, which have integration of motor neurons, would exhibit great utility in the understanding of neuromuscular diseases and as a preclinical test bed to study the use of neuromuscular pharmaceuticals. In this study we have generated such a model using primary cells cocultured in a 3D fibrin-based hydrogel which exhibits characteristics indicative of NMJ formation and enhanced myotube maturation, and presents a significant step forward in the formation of fully integrated engineered skeletal muscle.
